# Democracy, gender equality, advanced civil rights, economic welfare, and equitable distribution of wealth are related to the success of tobacco control

**DOI:** 10.18332/tid/218298

**Published:** 2026-04-30

**Authors:** Osman Elbek, Ahmet N. Emecen, Pelin D. Çetinkaya, Pelin P. Deniz, Oğuz Kılınç

**Affiliations:** 1 Süreyyapaşa Chest Diseases and Chest Surgery Training and Research Hospital, İstanbul, Türkiye; 2 Public Health Department, Faculty of Medicine, Epidemiology Subsection, Dokuz Eylul University, İzmir, Türkiye; 3 Department of Respiratory Disease, Cukurova University Faculty of Medicine, Adana, Türkiye; 4 Chest Diseases Department, Faculty of Medicine, İzmir University of Economics, İzmir, Türkiye

**Keywords:** social determinants of health, MPOWER, democracy, social rights, smoking prevalence

## Abstract

**INTRODUCTION:**

It is well known that there are many social and economic factors influencing tobacco use. There are very few studies addressing these aspects of the issue. Nevertheless, smoking is closely related to social, economic, cultural, and political factors. This study aims to present the aspects of the MPOWER application that should be expanded in order to achieve a more effective tobacco control policy.

**METHODS:**

Thirty-one countries were included in this secondary data analysis. The smoking prevalence of the countries in 2020 and the changes in smoking prevalence between 2010 and 2020 were taken as dependent variables. Criteria indicating the level of development, democratic standards, gender equality, civil rights, and basic health level of the countries were considered independent variables. Pearson (r) or Spearman (ρ) correlation analyses were conducted based on normality assumptions.

**RESULTS:**

There was a positive linear correlation between smoking prevalence and the total unemployment rate (ρ=0.78), income inequality (ρ=0.72), the Global Rights Index (ρ=0.54), and the Gender Inequality Index (ρ=0.45). In contrast, smoking prevalence was negatively correlated with current health expenditures (ρ= -0.55), the Human Development Index (ρ= -0.55), the Corruption Perceptions Index (r= -0.51), the World Justice Index (ρ= -0.47), the share of seats in parliament (r= -0.43), the Democracy Index (ρ= -0.41), and MPOWER (2008) (ρ= -0.38). There was a strong positive correlation between the percent change in smoking prevalence and income inequality (ρ=0.77), the total unemployment rate (ρ=0.64), the Gender Inequality Index (ρ=0.46), and the Global Rights Index (ρ=0.44). Conversely, the percent change in smoking prevalence was negatively correlated with the Freedom Index (ρ= -0.67), the Democracy Index (ρ= -0.67), the Human Development Index (ρ= -0.59), the Corruption Perceptions Index (r= -0.56), current health expenditure (ρ= -0.55), GDP per capita (ρ= -0.53), and MPOWER (2008) (ρ= -0.47).

**CONCLUSIONS:**

Besides the MPOWER application, factors correlated with the success of tobacco control are social determinants of health, gender equality, and advanced democracy.

## INTRODUCTION

The World Health Organization (WHO) announced the MPOWER criteria (Monitoring tobacco use and prevention policies; Protecting people from tobacco smoke; Offering help to quit tobacco use; Warning about the dangers of tobacco; Enforcing bans on tobacco advertising, promotion and sponsorship; Raising taxes on tobacco), consisting of six components, for implementing tobacco control in 2008, five years after the adoption of the Framework Convention on Tobacco Control^1^. Türkiye established the National Tobacco Control Program in 2008^2^ and became the first and only country in the world to implement all six components in 2013^3^. This success and leadership of Türkiye in the field of tobacco control was supported by WHO with the Anti-Smoking Award (2008), the WHO European Region Anti-Tobacco Award (2010), the WHO 2012 World No Tobacco Day Special Award (2012), the Special Prestige Award for Global Tobacco Control presented by WHO Director-General Margaret Chan (2013), and finally the WHO 31 May World No Tobacco Day Award (2021). Moreover, the success of Türkiye was both specially announced in the annual WHO reports under the headline ‘Türkiye marks singular achievement in tobacco control’^3^ and reinforced in a book called ‘Story of Commitment and Leadership’ published by the Regional WHO Office for Europe^4^.

As a matter of fact, the rate of tobacco use dropped by 13.4% between 2008 and 2012 in Türkiye – a favorite country of the WHO in the field of tobacco control and a model for other countries to follow for its success^5^. Similarly, the rate of smoking before the age of 15 years dropped from 19.6% to 16.1%, the rate of passive exposure in restaurants dropped from 55.9% to 12.9%, and the rate of considering quitting smoking rose by 14.4%^5^. However, the results of the Global Adult Tobacco Survey conducted in 2016 showed that the tobacco use rate in Türkiye increased for both males and females between 2012 and 2016. This increase was 6% in males and 47% in females. Additionally, when considering individuals who smoke, the rate of considering quitting smoking decreased from 55.2% to 32.8%^5^, and the smoking prevalence increased by 4.5% between 2012 and 2016^6^. In 2022, the findings of the health survey held by Türkiye Statistical Institute confirmed that the rate of tobacco use among individuals aged ≥15 years in Türkiye had risen from 28% in 2019 to 28.3% in 2022^7^.

This failure in Türkiye, which has been set as a model by the World Health Organization as a ‘leading country’ in the field of tobacco control after 2012, draws attention due to the integration of all components of the MPOWER application into the national law. However, the literature suggests that MPOWER measures are effective in reducing smoking prevalence, consumption, and deaths related to tobacco use^8-11^. Nevertheless, the failure of Türkiye in the recent period raised questions about the effectiveness of MPOWER applications.

The scientific literature contains few studies that examine the impacts of social and economic factors affecting tobacco use based on populations other than MPOWER measures^12^. However, smoking is closely associated with social, economic, cultural, and political factors. Therefore, the problems experienced in Türkiye may be due to the exclusion of MPOWER components in social determinants. On the other hand, a study that investigated the response of Türkiye, Brazil, and South Korea to the COVID-19 pandemic showed that the state capacities and populist administrations of the countries were the key factors affecting the outcome of the pandemic in the field of health^13^. A similar situation may be valid for the area of tobacco use. This is a political issue of tobacco control, which is related to states’ capacity and governance patterns. This study aims to investigate the correlation between the MPOWER package, the ‘social determinants of health’ criteria frequently stressed by the WHO in recent years without remaining specific to Türkiye, and the capacity and democratic attitudes of the administrative structures of countries with tobacco control, and thus to foresee the aspects of the MPOWER policy intervention that should be expanded in order to achieve a more effective tobacco control.

## METHODS

### Data and sample

The data of 31 countries with diverse characteristics regarding tobacco consumption and control (United States, Argentina, Australia, Bangladesh, United Kingdom, Brazil, China, Denmark, Indonesia, Philippines, France, India, Netherlands, Italy, Spain, Iceland, Japan, Canada, Qatar, Mauritius, Mexico, Norway, Russia, Chile, Saudi Arabia, Türkiye, Vietnam, New Zealand, and Greece) were included in the secondary data analysis, of ecological model design. Data on the countries’ implementation of the MPOWER package and tobacco usage levels were obtained from the websites of the World Health Organization and the World Bank^14-16^.

### Calculation of MPOWER score for countries

In 2008, the World Health Organization (WHO) introduced the ‘MPOWER policy package’, a set of six measures designed to counter tobacco companies’ market-creation strategies and control the spread of tobacco use. The acronym MPOWER represents the first letters of these six measures^17^. Data from the WHO 2010 and 2020 reports were used to assess the extent to which the 31 countries included in this study implemented the MPOWER package. Each implemented measure was assigned a score of one, with a maximum score of six for the full implementation of all measures.

### Dependent variables

The dependent variables were defined as each country’s smoking prevalence rates in 2010 and 2020 and the percent change (%) in smoking prevalence from 2010 to 2020 [(2020 prevalence - 2010 prevalence)/2010 prevalence]. Data for each country were obtained from the 2010 and 2020 reports of the World Bank (WB) and World Health Organization (WHO)^14-16^.

### Independent variables

The following indicators were included as independent variables: Maternal mortality ratio, Gender Inequality Index, Democracy Index, Income inequality (GINI coefficient), Gross domestic product (GDP) per capita, World Justice Project Index, the Human Development Index (HDI), Total unemployment rate, Share of seats in parliament, Labor force participation rate, Global Rights Index, Proportion of the population with at least some secondary education, Freedom Index, Current health expenditure as a percentage of GDP, Annual growth rate, and Corruption Perceptions Index. All data were freely available online and accessed from publicly available sources^18-33^.

### Statistical analysis

Normality was assessed using histograms and the Shapiro-Wilk test. Based on normality assumptions, Pearson or Spearman correlation analyses were conducted to examine the linear relationships between socio-economic, health, and governance factors and smoking prevalence, as well as the percent change in prevalence. The strength of the correlation coefficients (Pearson: r, Spearman: ρ) was interpreted according to Evans’ (1996) classification: very weak (0.00–0.19), weak (0.20–0.39), moderate (0.40–0.59), strong (0.60–0.79), and very strong (0.80–1.00). Statistical significance was defined as a two-tailed p<0.05. All analyses were performed using R version 4.3.1 (R Foundation for Statistical Computing, Vienna, Austria; https://www.R-project.org/).

## RESULTS

This analysis included 31 countries. Table 1 presents the correlation analysis results between countries’ indicators and smoking prevalence in 2020. There was a strong positive linear relationship between the total unemployment rate and smoking prevalence (WHO: ρ=0.78, p=0.003; WB: ρ= 0.80, p=0.002). Similarly, income inequality and the Global Rights Index were positively correlated with smoking prevalence according to WHO (ρ=0.72, p=0.03 and ρ =0.54, p=0.002; respectively). An increase in the population with at least some secondary education had a moderate negative correlation with smoking prevalence (WHO: r= -0.58, p=0.001; WB: r= -0.48, p=0.007). Additionally, current health expenditure (% of GDP), the Human Development Index, the Corruption Perceptions Index, and the World Justice Project Index were all negatively correlated with smoking prevalence, according to both WHO and WB data.

**Table 1:** Correlation analysis between countries’ indicators and smoking prevalence (2020) according to the World Health Organization and World Bank

*Indicators*	*Number of countries* *(N=31)* *n*	*WHO*		*World Bank*	
		*Correlation coefficient*	*p*	*Correlation coefficient*	*p*
Total unemployment rate (2019)	12	0.78^a^	0.003	0.80^a^	0.002
Income inequality (2019)	9	0.72^a^	0.030	0.65^a^	0.058
Global Rights Index (2020)	29	0.54^a^	0.002	0.40^a^	0.031
Gender Inequality Index (2020)	30	0.45^a^	0.012	0.33^a^	0.077
Maternal mortality ratio (2020)	30	0.35^a^	0.060	0.24^a^	0.209
Annual growth rate (2020)	30	0.34^2^	0.063	0.42^b^	0.021
Population with at least some secondary education (2020)	30	-0.58^b^	0.001	-0.48^b^	0.007
Current health expenditure (% of GDP)	31	-0.55^a^	0.001	-0.45^a^	0.011
Human Development Index (2020)	31	-0.55^a^	0.001	-0.43^a^	0.016
Corruption Perceptions Index (2020)	30	-0.51^b^	0.004	-0.42^b^	0.022
World Justice Project Index (2020)	27	-0.47^a^	0.013	-0.39^a^	0.045
Share of seats in parliament (2020)	30	-0.43^b^	0.018	-0.30^b^	0.113
Democracy Index (2020)	30	-0.41^a^	0.024	-0.22^a^	0.244
Labor force participation rate (2020)	30	-0.38^b^	0.037	-0.34^b^	0.062
MPOWER (2008)	30	-0.38^a^	0.038	-0.30^a^	0.104
GDP per capita (2020)	31	-0.37^b^	0.041	-0.22^b^	0.245
Freedom Index (2020)	31	-0.26^a^	0.158	-0.11^a^	0.546
MPOWER (2010)	31	-0.21^a^	0.265	-0.10^a^	0.598
MPOWER (2020)	31	-0.16^b^	0.189	-0.11^b^	0.542

a Spearman correlation coefficient (ρ).

b Pearson correlation coefficient (r).

According to WHO data, there was a negative, weak linear relationship between MPOWER (2008) and smoking prevalence (ρ= -0.38, p=0.038). However, MPOWER scores for 2010 and 2020 showed no significant relationship with smoking prevalence (Supplementary file Figure 1).

Table 2 summarizes the correlation analysis between countries’ indicators and the percent change in smoking prevalence from 2010 to 2020, according to the WHO. A positive correlation was observed between the percent change in smoking prevalence and factors such as income inequality (ρ=0.77, p=0.016), total unemployment rate (ρ=0.64, p=0.024), Gender Inequality Index (ρ=0.46, p=0.010), Global Rights Index (ρ=0.44, p=0.018), and maternal mortality ratio (ρ=0.43, p=0.018). In contrast, factors such as Freedom Index (ρ= -0.67, p<0.001), the Democracy Index (ρ= -0.67, p<0.001), the Human Development Index (ρ= -0.59, p<0.001), and the Corruption Perceptions Index (r= -0.56, p=0.001) were negatively correlated with changes in smoking prevalence (Table 2).

**Table 2:** Correlation analysis between countries’ indicators and the percent change in smoking prevalence (2010–2020) according to the World Health Organization

*Indicators*	*Number of countries* *(N=31)* *n*	*Correlation coefficient*	*p*
Income inequality (2019)	9	0.77^a^	0.016
Total unemployment rate (2019)	12	0.64^a^	0.024
Gender Inequality Index (2020)	30	0.46^a^	0.010
Global Rights Index (2020)	29	0.44^a^	0.018
Maternal mortality ratio (2020)	30	0.43^a^	0.018
Annual growth rate (2020)	30	0.04^b^	0.850
Freedom Index (2020)	31	-0.67^a^	<0.001
Democracy Index (2020)	30	-0.67^a^	<0.001
Human Development Index (2020)	31	-0.59^a^	<0.001
Corruption Perceptions Index (2020)	30	-0.56^b^	0.001
Current health expenditure (% of GDP)	31	-0.55^a^	0.001
GDP per capita (2020)	31	-0.53^b^	0.002
Population with at least some secondary education (2020)	30	-0.52^b^	0.003
World Justice Project Index (2020)	27	-0.52^a^	0.006
Share of seats in parliament (2020)	30	-0.49^b^	0.006
MPOWER (2008)	30	-0.47^a^	0.010
Labor force participation rate (2020)	30	-0.26^b^	0.158
MPOWER (2010)	31	-0.34^a^	0.061
MPOWER (2020)	31	-0.18^b^	0.332

a Spearman correlation coefficient (ρ).

b Pearson correlation coefficient (r). Percent change in smoking prevalence: (2020 prevalence - 2010 prevalence)/2010 prevalence.

Countries with higher MPOWER scores in 2008 tended to have a decrease in smoking prevalence from 2010 to 2020 (ρ= -0.47, p=0.010). Higher MPOWER scores in 2010 were weakly associated with decreased smoking prevalence from 2010 to 2020 (ρ= -0.34, p=0.061) (Supplementary file Figure 2).

## DISCUSSION

This study shows that MPOWER applications were by no means the only factor associated with tobacco use; on the contrary, ‘social determinants of health’ criteria and the governance patterns of nation-states are also key components related to tobacco control.

The present study indicated that societies that live with limited social and political rights in countries where unemployment rates are high, income inequality among citizens is high, and yet an advanced democracy has not developed, were more likely to smoke cigarettes. On the other hand, smoking prevalence has dropped, and cessation rates have risen in countries that have expanded their populations with secondary educational levels and alleviated gender inequality due to the welfare model of the state. In this context, as a reflection of gender equality, the rise in the number of women in parliament and women’s participation in the workforce is linearly correlated with a drop in cigarette use.

It is known that low socio-economic status is correlated with high personal tobacco consumption. While high unemployment rates and low incomes lead to an upsurge in personal consumption, ownership of property, and lack of worry about subsistence are correlated with the non-use of tobacco products in both genders^34^. There is also a positive correlation between low socio-economic status and heavy smoking^35^. In this sense, socio-economic disadvantage that develops depending on income inequality leads to both higher smoking prevalence and more cigarette consumption in both genders^35^. A similar correlation is also valid for education background. A study conducted in Ireland found that those with the lowest education level and those who lived in the most impoverished regions had the highest rate of daily cigarette smoking, and this inequality between social strata intensified and deepened over time^36^. Lower awareness of the health hazards associated with smoking, more nicotine addiction, and a less supportive social environment are some factors that explain this socio-economic difference^34^. On the other hand, segments with low socio-economic status constitute the target group for the tobacco industry due to their heavy population density. Given this framework, it is remarkable that a study conducted in Finland found a significantly lower probability of identifying tobacco sellers in regions of the city with high average income and a higher probability in regions with the lowest income category^37^.

There is generally a paradoxical correlation between gender inequality and individual tobacco consumption. A study conducted in Germany using the United Nations Gender Inequality Index showed that while gender inequality decreased across the country from 1960 to 2005, female smoking prevalence grew and became closer to that of the male gender^38^. The data indicate that the reduced gender inequality based on individual consumption raises smoking rates, especially among people with higher levels of education^38^. However, the findings of the present study suggest that this correlation, which is valid on an individual basis, becomes invalid on a country and population basis. This is because socio-economically developed countries that have alleviated gender inequality have improved welfare and alleviated inequalities, especially economic inequalities between social strata. Such countries are capable of effectively using both MPOWER and non-MPOWER factors affecting tobacco use due to the positive impact of advanced democracy and social rights. On the other hand, the present study may not have shown the rise in use by female gender in countries that have alleviated gender inequality since it analyzed the impact on the consumption of the total population without analyzing male and female gender groups separately.

Income, education level, and gender inequality are the most important social determinants that affect tobacco use on both individual and population basis. Therefore, tobacco control policies in countries should primarily target the most vulnerable and fragile segments of society with the highest smoking prevalence rates^39^. Public policies that aim to eliminate existing inequalities and bring everyone to equality in health also mean an effective intervention for tobacco control. However, there are very few studies on the impacts of social determinants of health on tobacco control and what aspects of MPOWER should be improved. One of these studies was conducted by Watson et al.^12^, as the first and only systematic review of population-level determinants of national tobacco consumption. This systematic review showed that countries’ economic status, educational policies, macro-economic factors, unemployment rates, and the level of social welfare are factors affecting tobacco use other than MPOWER, just as we found in the present study. The major disadvantage of this systematic review is its reliance on data from Western Europe, particularly the United Kingdom, Germany, Ireland, Spain, France, Italy, Greece, the Netherlands, and Sweden^12^. On the other hand, the data of the present study covers Argentina, Australia, Bangladesh, Brazil, Canada, Chile, China, Japan, India, Indonesia, New Zealand, the Philippines, Qatar, Mauritius, Mexico, Russia, Saudi Arabia, Türkiye, the United States of America, and Vietnam, in addition to Western European countries.

The findings of the present study suggest that economic development (lower unemployment rate, higher HDI) and lower economic inequalities reduce smoking rates and increase cessation rates, as shown in the systematic review by Watson et al.^12^. Likewise, data from the present study indicated that educational policies helped reduce cigarette consumption, as the aforementioned research suggests. In addition, the data, as a unique conclusion, suggested that democratic standards of countries and gender were also factors affecting tobacco use. The findings of the present study showed that as the democratic standards of countries improve, social freedoms increase and civil rights become more empowered, the perception of corruption reduces, the rule of law develops, gender inequality is alleviated, the rate of elected female parliamentarians increases, cigarette consumption in society drops, and the cessation rate rises. Therefore, tobacco control policies should be integrated into the economic, educational, democratic, and egalitarian public policies of countries. Governments should develop their own democratic governance policies and establish the rule of law in their countries through public programs integrated with tobacco control.

The present study indicated that the smoking rate was significantly negatively correlated with some basic health measures, such as the increase in maternal mortality rate, and positively associated with economic resources allocated to health services. In our opinion, this correlation corresponds to a condition that progresses together rather than showing a causal relationship. This is because countries that can allocate high economic resources to health services are often developed countries. Such countries have good health metrics, such as maternal mortality rate, due to their socio-economic development and the competence of the health services they provide to their citizens. Considering that rising economic prosperity, equitable sharing of social welfare, and gender equality are negatively correlated with cigarette smoking – one of the main findings of the present study – it is expected that the population in developed countries with good basic health measures would have low smoking rates and high cessation rates. In this sense, it can be predicted that countries that can effectively implement tobacco control policies in the world actually have developed and competent health systems in general.

Data from the literature show that high MPOWER scores in countries are positively correlated with smoking cessation^40^. Likewise, high MPOWER scores ensure effective tobacco control by means of blocking tobacco advertisements, warning individuals about its harms, assisting individuals to quit smoking, and raising taxes^41^. Husain et al.^8^ showed that a rise of 1 point in MPOWER scores was associated with a drop of 0.39 points in countries with a high smoking prevalence and a drop of 0.50 points in countries with a low smoking prevalence. On the other hand, a systematic analysis that reviewed 62 studies revealed a correlation between MPOWER measures and 99 out of 155 effective factors at the population level, and that 55 of these measures led to a decrease in smoking prevalence^12^. This study found that increased cigarette prices and higher taxes were highly effective in reducing smoking, while the impact of other MPOWER policies was limited^12^. The present study also found significant correlations between the MPOWER scores of countries in 2008 and the smoking prevalence in 2020, and between the MPOWER scores in 2008 and 2010 and the change in smoking prevalence between 2010 and 2020. Data from the present study showed that the higher the MPOWER scores of countries in 2008, the less the population of that country smoked in 2020, and the higher the MPOWER scores in 2008 and 2010, the more favorable the decrease in smoking prevalence between 2010 and 2020.

### Limitations

The most important limitation of this study is the openness of the cause/effect relationship to discussion due to the nature of the ecological study. Additionally, due to the unavailability of detailed data for all countries worldwide, the study includes a relatively small sample size. Caution should be exercised when interpreting the results and inferring causal relationships due to this limitation. Future studies should involve an increased amount of country-level data and include research on the field effectiveness of tobacco control interventions. On the other hand, another limitation is the lack of documentation of the impacts of MPOWER and non-MPOWER factors on specific groups, such as females and adolescents, while the effects of MPOWER and non-MPOWER factors on the population as a whole were documented. In our opinion, these limitations provide a suitable baseline for further research. On the other hand, the present study provides an opportunity to expand the existing MPOWER policy by analyzing the social, economic, and cultural factors that play a role in tobacco use on a population basis, a topic that is not frequently researched in the literature. This study also draws attention to the close correlation of tobacco control practices with the democratic governance of states, expanded social freedoms, improved civil rights, and gender equality policies.

## CONCLUSIONS

Findings of the present study highlight the requirement to implement public policies that not only lower unemployment and improve public welfare but also expand social freedoms by raising the democratic standards of states, promoting citizens’ ability to claim their rights, and striving for both economic and gender equality throughout society, in addition to MPOWER measures, to achieve competent tobacco control.

## Supplementary Material



## Data Availability

The data supporting this research are available from the authors on reasonable request.
