# Supplementary material for: Democracy, gender equality, advanced civil rights, economic welfare, and equitable distribution of wealth are related to the success of tobacco control
Source: Tob Induc Dis. 2026 Apr 30;24:10.18332/tid/218298. doi: 10.18332/tid/218298 (PMC13135247; doi:10.18332/tid/218298)
Supplement: Supplementary file 1 [file TID-24-53-s1.pdf]

Supplementary file

Supplementary Figure 1. Correlation between MPOWER and smoking prevalence according to the World Health Organization and World Bank for 2020. Dots show the countries included in the analysis. Dotted and solid lines represent regression lines derived from linear regression analysis

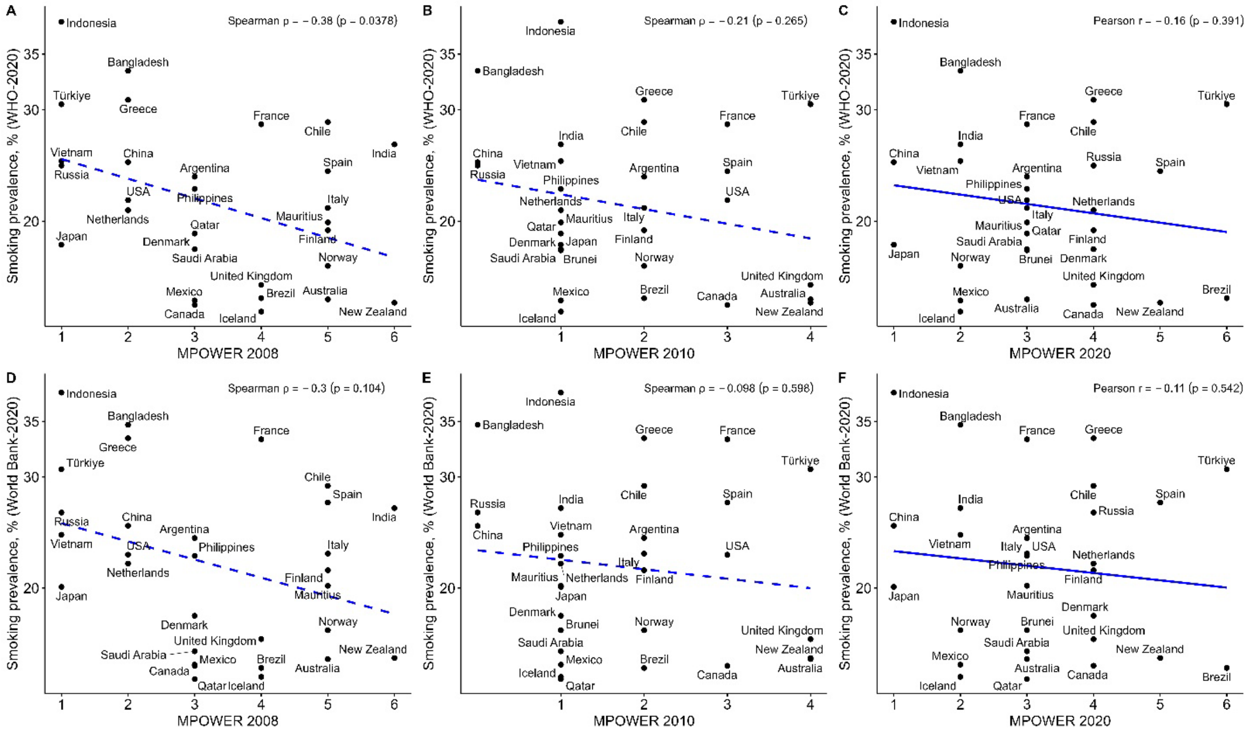

Supplementary Figure 2. Correlation between MPOWER and percent change of smoking prevalence from 2010 to 2020 according to the World Health Organization. Dots show the countries included in the analysis. Dotted and solid lines represent regression lines derived from linear regression analysis

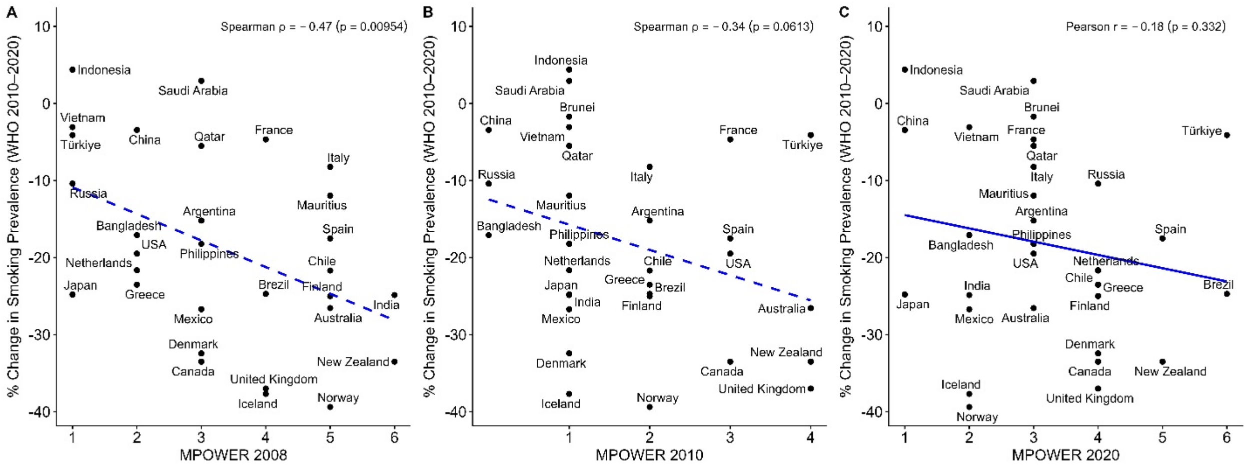

© 2026 Elbek O. et al.
